# Transcriptome Profile Changes Associated With Heat Shock Reaction in the Entomopathogenic Nematode, *Steinernema carpocapsae*

**DOI:** 10.3389/fphys.2020.00721

**Published:** 2020-07-10

**Authors:** Yi-Fei Xie, Xiu-Dan Wang, Wu-Hong Zhong, Dao-Hong Zhu, Zhen He

**Affiliations:** ^1^Hunan Academy of Forestry, Changsha, China; ^2^College of Life Science, Central South University of Forestry and Technology, Changsha, China

**Keywords:** *Steinernema carpocapsae*, transcriptome, heat shock response, differentially expressed gene, entomopathogenic nematode

## Abstract

The entomopathogenic nematode *Steinernema carpocapsae* has been used for control of soil insects. However, *S. carpocapse* is sensitive to environmental factors, particularly temperature. We studied an *S. carpocapse* group that was shocked with high temperature. We also studied the transcriptome-level responses associated with temperature stress using a BGIseq sequencing platform. We *de novo* assembled the reads from the treatment and control groups into one transcriptome consisting of 43.9 and 42.9 million clean reads, respectively. Based on the genome database, we aligned the clean reads to the Nr, Gene Ontology (GO), and Kyoto Encyclopedia of Genes and Genomes (KEGG) databases and analyzed the differentially expressed genes (DEGs). Compared with the control, the heat-shocked group had significant differential expression of the heat shock protein (HSP) family, antioxidase [glutathione S-transferases (GSTs) and superoxide dismutase (SOD)], monooxygenase (P450), and transcription factor genes (DAF-16 and DAF-2). These DEGs were demonstrated to be part of the Longevity pathway and insulin/insulin-like signaling pathway. The results revealed the potential mechanisms, at the transcriptional level, of *S. carpocapsae* under thermal stress.

## Introduction

Traditional chemical pesticides are used to reduce agricultural losses caused by insect pests. However, chemical pesticide residues pose hazards to both humans and the environment (Thomas, 2018). Biological control can be an effective method to reduce pesticide residues, and entomopathogenic nematodes (EPNs) can be useful biocontrol agents. EPNs can help manage damaging pests in many farming systems (Cruz-Martinez et al., 2017; Dlamini et al., 2019), and they have been commercialized in many countries. The EPN *Steinernema carpocapsae* interacts with symbiotic bacteria (*Xenorhabdus nematophila*) and parasitizes soil insect pests (Richards and Goodrich-Blair, 2009; Labaude and Griffin, 2018). Nematodes in the infective juvenile stage (dauers) search for insect hosts in the soil and enter through wounds or natural orifices, such as the mouth, spiracle, and anus (Dlamini et al., 2019). The symbiotic *Xenorhabdus* bacteria are released into the insect hemocoel and propagate *in vivo* (Khandelwal and Banerjee-Bhatnagar, 2003; Khandelwal et al., 2004). Also, a toxic venom protein is directly synthesized in *S. carpocapse* (Balasubramanian et al., 2010) and released *in vitro* (Lu et al., 2017). This is a new component of the lethal mechanism of *S. carpocapsae*. *S. carpocapsae* plays a key role in delivering the toxins to the host during pathogenesis (Chang et al., 2019). *S. carpocapsae* are sensitive to many environmental factors, such as temperature, UV, and humidity (Kepenekci et al., 2015; Salame and Glazer, 2015; El-Borai et al., 2016). It is probable that temperature is the most important factor impacting the efficacy of EPNs (Odendaal et al., 2016; Wilson et al., 2016). Therefore, understanding how *S. carpocapsae* resists thermal stress may help improve the ability of EPNs to serve as biological control agents.

The transcriptome can be used to study the responses of an organism, at the transcriptional level, following experimental treatments. In nematodes, toxic chemicals activate the immune response and can change their fecundity and life span. RNA-Seq analyses demonstrated that some differentially expressed genes (DEGs) were related to the immune response (Martis et al., 2017; Di et al., 2018). Changes of gene expression, in different life cycles, were detected by transcriptome sequencing (Seiml-Buchinger and Matveeva, 2019). The transcriptomes and genomes of *Steinernema* spp. have been characterized (Dillman et al., 2015; Rougon-Cardoso et al., 2016; Yaari et al., 2016; Serra et al., 2019). However, no studies have focused on the specific genes and pathways involved in the heat shock reactions of *S. carpocapsae*.

We studied changes in the transcriptome related to heat shock treatment and analyzed the pathways and genes related to different temperature treatments. This study demonstrates the heat shock response mechanisms of *S. carpocapsae* at the molecular level. The results may promote the practical use of *S. carpocapsae* in biocontrol.

## Materials and Methods

### Nematode Cultures

The *S. carpocapsae* (strain All) was supplied by the Guangdong Institute of Applied Biological Resources, Guangdong Province, China (Han et al., 1997). We used *Galleria mellonella* larvae as the hosts of *S. carpocapsae* (Kaya and Stock, 1997). The infected hosts were cultured in a 25°C incubator. These nematodes were selected as the controls and original nematodes for the heat shock tests. After the culture period, the infective juveniles (IJs) were introduced into sterile distilled water in a culture dish in preparation for the next step.

### Heat Treatment and Sample Collection

We measured the nematode heat resistance based on procedures used in a previous study (Yaari et al., 2016). We exposed the nematodes to 32°C for 3 h. They were then transferred to 23°C for 1 h to recover and finally exposed to 35°C for 5 h. We then selected 10 drops with 10-μl suspensions in each drop and calculated the average nematode mortality. Each drop contained 80–100 nematodes. The surviving nematodes were cultured in the host in normal condition, and the offspring were tested in the same manner. As the survival rate exceeded 50%, the high temperature used in the experiment was increased from 35 to 38°C with successive generations of nematodes. The nematodes whose survival rate exceeded 50% at 38°C were collected as the treatment group. Samples for RNA extraction were taken from all instars. Both the heat shock treatment and the control group were prepared in three replicates. Approximately 2,000–2,200 nematodes, representing all growth stages, were collected for each replicate.

### RNA Isolation and Transcriptome Sequencing

Total RNA for sequencing was isolated using the RNeasy^®^ plus Micro Kit according to manufacturer’s instructions (Qiagen, Hilden, Germany). A NanoVue spectrophotometer (GE Healthcare Bio-Science, Uppsala, Sweden) was used to assess the quality of total RNA. The degradation and quality of the RNA were tested by electrophoresis on a 1.0% agarose gel. To isolate poly(A) mRNA, magnetic beads with oligo (dT) were used. After the fragmentation of the mRNA under the effect of the fragmentation buffer, the cDNA was synthesized using the template mRNA fragments. These short cDNA fragments were resolved and purified by ethidium bromide buffer for end reparation and connected with adapters. After the cDNA samples were selected for PCR amplification and quantified and qualified using an Agilent 2100 Bioanaylzer and ABI StepOnePlus Real-Time PCR System (Agilent Technologies, Santa Clara, CA, United States). The library was sequenced using an BGIseq PE150 system (Shenzhen, China). The cDNA library construction and sequencing were performed by Beijing Genomics Institution (Shenzhen, China).

### *De novo* Assembly and Bioinformatics Analysis

The adaptors and low-quality sequences from the raw reads were removed using FASTX^1^. SOAP2 (version 2.2.1)^2^ was used to filter out potential rRNA reads, and the data were filtered to be clean reads. The filtered clean reads were mapped to the reference genome, *S. carpocapsae*, using HISAT2 (Hierarchical Indexing for Spliced Alignment of Transcripts)^3^ (Rougon-Cardoso et al., 2016). The assembled *de novo* transcriptome was implemented using Trinity (Grabherr et al., 2011). The reads with overlap length were combined to structure the contigs and then mapped back to contigs. Trinity connected the contigs from the same transcript that could not be extended on either end and formed the defined unigenes. The unigenes obtained were distributed into two groups: singletons and clusters. With an *E*-value cutoff of 10^–5^, the sequences were compared based on the databases of Nr and Kyoto Encyclopedia of Genes and Genomes (KEGG) database. The software Blast2GO was used to analyze the gene annotation with Gene Ontology (GO) terms^4^ with default parameters. The KEGG database was used to perform pathway annotation analysis^5^. Selected from the Nr annotation, the interesting genes were analyzed using NCBI BLASTx^6^.

### Analysis of the Differential Gene Expressions

After the clean reads were aligned to the genome sequence, the expression level of each sample was calculated using RSEM (Li and Dewey, 2011) based on the RPKM (Reads Per Kilobase per Million mapped reads) value. Next, significance levels (*P*-values) of DEGs between treatment and control groups were calculated using edgeR (Robinson et al., 2010), an R package^7^ recommended for use in cases without biological or technical replicates. According to the recommendation of the edgeR manual^8^, all of the parameters were set. By controlling the false discovery rate (FDR), two methods were used to correct significant levels (*P*-values): the Benjamini and Hochberg and Storey and Tibshirani methods (Benjamini and Hochberg, 1995; Storey and Tibshirani, 2003). For the accuracy of the improved DEGs, we defined the fold change of gene expression ≥ 2 and adjusted the *P* ≤ 0.001 as the criteria for significant differences in gene expression levels. To deeply extract the DEGs and pathway data that were related to the treatment and control, GO and KEGG pathway cluster analyses were conducted using Blast2GO and KOBAS, respectively. After revision by FDR, the notable levels were defined as those with a *P* ≤ 0.001.

### Substantiation of the RNA-Seq Data and Gene Expression by Quantitative Real-Time PCR

To convey the results of the transcriptome, quantitative real-time PCR (qPCR) was conducted for the key genes in the heat reaction, including SOD and the heat shock proteins (HSPs). Following alignment using NCBI BLASTx, the isolated sequences were preliminarily confirmed, and primers of these genes are designed using Primer 3.0^9^ and delineated in Supplementary Table S2. The expression abundances of mRNA were detected by qPCR using ABI StepOne Plus^TM^ Real-Time PCR System (Life Technologies, Woodlands, Singapore) with GoTaq^®^ qPCR master mix (Promega, Madison, WI, United States). The templates for qPCR were the same as those for the RNA-Seq. qPCR detection was based on a 20-μl reaction, composed of 10 μl master mix, 7 μl ddH_2_O, 1 μl template cDNA, and 1 μl of each primer. The cycling program is as follows: 95°C for 2 min, 40 cycles of 95°C for 15 s, and 60°C for 30 s, 60°C for 15 s, and 95°C for 30 s. The relative expression was calculated using the 2^–Δ^ ^Δ^ ^Ct^ method (Pfaffl, 2001) with Actin set as the reference gene (Yan et al., 2011). Fold changes were clarified after the relative expression values were standardized by the lowest value.

## Results

### Isolation of the Differentially Expressed Genes Between the Heat-Shock Treatment and Control

The raw data were filtered after sequencing, and the low-quality reads were discarded. We obtained 43.9 and 42.9 million clean reads with an average of approximately 96.6 and 96.1% Q20 bases for the treatment and control groups, respectively. The filtered clean data were mapped to the *S. carpocapsae* genome. The percentage of mapping was 82.2% in the control and 88.9% in the treatment group. A total of 72.3% (15,850) and 65.5% (14,354) of all the genes (21,917) in the treatment and control groups were obtained as expressed genes (FPKM > 1). For functional annotation, the sequences were aligned to the database, including Nr, AnimalTFDB 2.0, GO, and KEGG. The number of annotated genes in the TFDB database was 2,206, as well as 13,319, 12,525, and 6,902 genes in the KEGG, Nr, and GO databases, respectively.

A total of 7,891 determined genes have been significantly expressed between the treatment and control groups, including 4,902 upregulated and 2,989 downregulated DEGs (Supplementary Figure S1). The top 10 most upregulated and downregulated genes are shown in Supplementary Table S1. Among them, the top three upregulated DEGs were ladderlectin-like protein, proline-rich extension-like protein, and synapse-associated protein. The three most downregulated DEGs were WD repeat domain 39, followed by decaprenyl-diphosphate synthase and lipase-like protein. In addition, some longevity regulating pathway and phosphoinositide 3-kinase (PI3K)-Akt signaling pathway genes were conspicuously expressed, such as insulin-like receptor and nematode cuticle collagen domain protein. Finally, several DEGs were detected as hypothetical or predicted proteins, indicating that their functions are not known and merit investigation.

### Gene Ontology and Kyoto Encyclopedia of Genes and Genomes Analysis of the Differentially Expressed Genes

**FIGURE 1:**
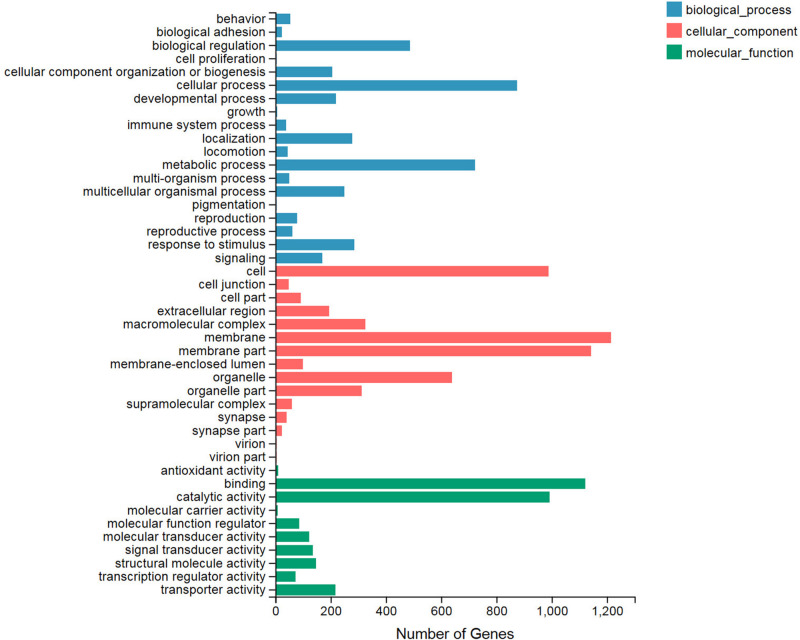
Gene Ontology (GO) functional enrichment analysis for the differentially expressed genes (DEGs). All of the GO terms are categorized into three clusters: biological process (blue), cellular component (red), and molecular function (green). The X axis indicates the number of DEGs. The Y axis represents the GO terms.

We classified significant enriched GO terms involved in the heat shock reaction by their determination as DEGs of the treatment and control groups. All of the DEGs were categorized into three GO types: biological process, cellular component, and molecular function, and these contained 19, 15, and 10 terms, respectively (Figure 1). In the biological process category, the top three terms with the largest number of DEGs included cellular process (874), metabolic process (723), and biological regulation (488). Among the cellular component category, the membrane (1,214), membrane part (1,143), and cell (988) terms contained the greatest number of DEGs. Binding, catalytic activity, and transporter activity were the top three terms in the molecular function category. Among all of these terms, the three least common terms were pigmentation (biological process), virion (cellular component), and virion part (cellular component), which all included four DEGs.

**FIGURE 2:**
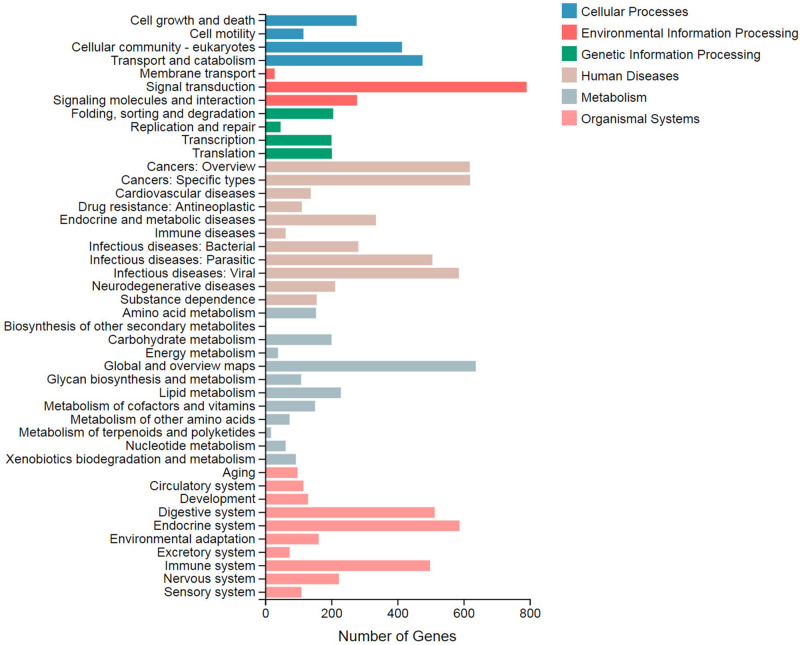
Statistics of the Kyoto Encyclopedia of Genes and Genomes (KEGG) classification of the differentially expressed genes (DEGs). The X axis displays the number of DEGs. The Y axis displays the second KEGG pathway terms. All of the second pathway terms are categorized to six pathway classifications indicated in different colors.

In the KEGG pathway enrichment analysis, 44 terms were classified to six categories: cellular processes, environmental information processing, genetic information processing, human diseases, metabolism, and organismal systems (Figure 2). Among them, signal transduction (791), global and overview maps (637), and cancers: specific types (620) were top three DEG terms. These terms were categorized to environmental information processing, metabolism, and human diseases, respectively. The term “biosynthesis of other secondary metabolites” included four DEGs, while the lipid metabolism term included 229 DEGs.

### Analysis of Heat Shock-Related Differentially Expressed Genes

Annotation of the DEGs showed that some genes were clearly associated with the heat shock response. After isolation, some DEGs were identified as heat shock-related DEGs (HRDs) to comprehensively understand the stress response of the HRDs to heat shock. Based on the preliminary categories of heat shock genes in nematodes in previous studies (Yaari et al., 2016), we isolated and identified four pathways: Longevity regulating pathway, IIS (insulin/insulin-like signaling) pathway, Drug metabolism-CYP450 pathway, and Fatty acid metabolism pathway (Table 1). Among the four pathways, 25 HRDs were selected, including 17 upregulated and eight downregulated. The top three most upregulated HRDs were Fatty acids protein 6 (8.3), Acyl-CoA synthetase (7.4), and LIPS17 (7.1). DAF-2 (-11.2) was the most downregulated HRD.

**TABLE 1:** Differentially expressed genes related to the heat shock responses.

**Pathway and gene ID**	**Annotation**	**Log2ratio (Treatment/Control)**	**Diff**	***P*-value**
**Longevity regulating pathway-worm**				
L596_026160	DAF-16	2.1	Up	6.64e-63
L596_030833	GCS-1	1.4	Up	2.66e-231
L596_011486	Forkhead	3.4	Up	0
L596_017052	RSK-1	−1.1	Down	0
L596_017665	LIPS17	7.1	Up	0
L596_015131	PARP	1.1	Up	7.05e-31
L596_014963	DVE1	−1.2	Down	9.06e-255
L596_018437	SOD	6.4	Up	9.17e-296
L596_021024	Hsp12.2	6.5	Up	0
L596_018763	Hsp12.6	3.9	Up	0
L596_026575	Hsp70	2.5	Up	0
L596_022153	Hsp20	2.1	Up	0
**Insulin/insulin-like signaling pathway**				
L596_018750	DAF-2	−11.2	Down	4.07e-146
L596_020446	Insulin receptor	1.4	Up	0
L596_024292	PDK1	−1.4	Down	0
L596_027363	EIF4E	−1.2	Down	1.47e-261
L596_017931	GSK	−1.2	Down	8.62e-9
L596_017052	RPS6K	−1.1	Down	0
**Drug metabolism-CYP450 pathway**				
L596_010034	CYP2C	2.4	Up	6.18e-123
L596_020572	GST	1.8	Up	0
**Fatty acid metabolism pathway**				
L596_024671	Fatty acids protein 6 (FAP6)	8.3	Up	0
L596_011626	Acyl-CoA synthetase	7.4	Up	0
L596_017359	Fatty acid desaturase	2.3	Up	0
L596_027870	Carnitine palmitoyl transferase	1.9	Up	9.94e-30

### Validation of the RNA-Seq Results by a Quantitative Real-Time PCR Analysis of 10 Heat Shock-Related Differentially Expressed Genes

**FIGURE 3:**
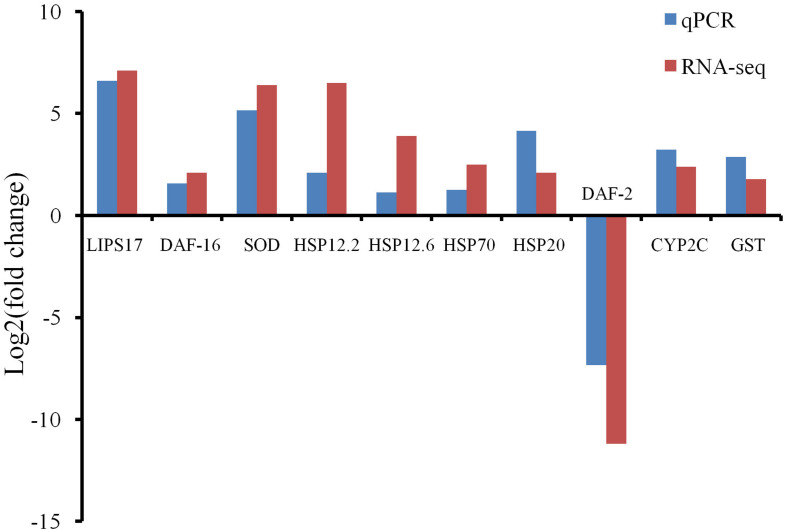
Quantitative real-time PCR (qPCR) detection for RNA-Seq results confirmation. Comparison of the RNA-Seq and qPCR results in expression level fold change of 10 selected heat shock-related differentially expressed genes.

Ten HRDs, which were clustered to different groups and key pathways, were isolated to validate the RNA-Seq results using qPCR. The expression trends of all 10 selected HRDs determined by qPCR were consistent with those detected by RNA-Seq, indicating that the transcriptome analysis was reliable (Figure 3).

## Discussion

Transcriptome technology has been used to obtain detailed information on the expression of genes in other species of nematodes. In *Bursaphelenchus xylophilus* and *Pelodera strongyloides*, the average clean reads of each treatment were 50.8 and 47.5 million, respectively (Wang et al., 2019; Zhang et al., 2019). In previous studies on the transcriptome of *S. carpocapsae*, nearly 15 million clean reads were obtained, and 95% of the reads were mapped to the genome using the Illumina platform. In addition, the 454 platform characterized 9,274 sequences in *S. riobrave* (Yaari et al., 2016). In our study, the mapping rates were 82.2% in the control and 88.9% in the treatment groups, which indicated the reliability of RNA-Seq. In *B. xylophilus* and *P. strongyloides*, the clean reads exceeded 42 million, which is far more than the number of reads documented in previous studies.

In *Caenorhabditis elegans*, alteration of lipid metabolism was involved in the stress reaction mechanisms (Zhang et al., 2010; Reis et al., 2011; Barros et al., 2012). When *C. elegans* became stressed, the distribution of nutrition can be affected in the nematode (Kelli et al., 2017). Some chemicals produced by plants can activate both fat consumption and heat stress tolerance, delineating the possible relationship between these two mechanisms (Hirano and Sakamoto, 2019). Although the organic compounds in lipid metabolism were not measured in this study, the DEGs in lipid metabolism and transportation were detected, suggesting a possible modification of nutrient allocation. It is not known if these changes affect energy redistribution.

Heat shock stress has often been studied. In *C. elegans*, the AFD neuron is the sensor that perceives increasing temperatures (Prahlad et al., 2008; Tatum et al., 2015). The thermal stress excites the FLP-6 neuropeptidergic signaling in the AFD neuron (Lee and Kenyon, 2009). The secretion of FLP-6 represses insulin-like peptide INS-7 and thus affects the downstream genes *via* the IIS pathway (Chen et al., 2016). INS-7 collaborates with DAF-2 in the downstream part of this pathway (Kawano et al., 2006; Sato et al., 2014). Silencing of DAF-2 stimulated DAF-16 expression and thus increased longevity in *C. elegans* (Murphy and Hu, 2013; Sonani et al., 2014). The KEGG analysis, based on our transcriptome data, indicated that the DAF-2 and DAF-16 were categorized in the IIS and Longevity regulation pathway. The expressions of *daf-2* and *daf-16* were found to be downregulated and upregulated, suggesting a negative regulation (repressor) relationship between these two genes. These results suggested that DAF-2 and DAF-16 could play a role in the upstream heat shock reaction in *S. carpocapsae*. These two factors will be studied using RNA interference in future studies.

GSTs enzymes are involved in the detoxification of metabolites. They are found in most species, including microorganisms, plants, and animals. In nematodes, GSTs enable detoxification of the endogenous chemicals secreted by the hosts (Brophy and Pritchard, 1994; Campbell et al., 2001; Espada et al., 2016; Matouskova et al., 2016). DAF-16 can upregulate the expression of GSTs and help extend the life span of *C. elegans* under thermal stress (Shanmugam et al., 2017). This demonstrated that GSTs are critical enzymes that protect the worms from a stressful environment. Similarly, thermal stress increased the expression of GSTs in *S. carpocapsae*, as well as the expression of DAF-16. Thus, GSTs, under the regulation of DAF-16, are likely involved in the heat shock reaction process.

Heat shock proteins are important enzymes that protect nematodes from protein denaturation at high temperatures (Kim et al., 2017). In the heat shock response, *hsps* are primarily controlled by two regulators, *heat shock factor-1* (*hsf-1*) and *daf-16*. In the early stages of nematode development, HSF-1 is negatively regulated by *daf-16*-dependent longevity genes (DDL-1&2), which belong to the IIS pathway (Chiang et al., 2012). In contrast, DAF-16 is modulated by DAF-2 in the adult stage (Volovik et al., 2012; Murphy and Hu, 2013). HSP12.6 and HSP16.2 are key enzymes in resistance to high temperatures (Kim et al., 2017; Hirano and Sakamoto, 2019). Moreover, HSP70 interacts with other proteins to recover protein stability *via* disaggregation (Nillegoda et al., 2015). The *hsps*, including *hsp12.2*, *hsp12.6*, *hsp70*, and *hsp20*, were upregulated in *S. carpocapsae* after the heat shock (Table 1), suggesting that these genes function in anti-thermal stress. A previous study revealed that the anti-thermal stress process in nematodes was activated by the neuron sensor AFD and finally operated by GSTs and HSPs.

In summary, we performed a comparative transcriptomic analysis of an *S. carpocapsae* line exposed to high-temperature treatment. Many DEGs were identified and transcriptionally validated to be key factors involved in the mechanism of anti-heat shock in *S. carpocapsae*. These included GSTs, P450s, HSPs, SODs, and transcription factor genes. Their roles and cooperative relationships, as well as the association between nutrient distribution and anti-thermal mechanisms, require further investigation. The gene expression profiling and differentially expressed identification responses related to heat shock tolerance in *S. carpocapsae* provide information for further functional analysis of the molecular mechanisms involved.

## Data Availability Statement

The datasets generated for this study can be found in the NCBI SRA accession PRJNA635371.

## Author Contributions

Y-FX, D-HZ, and ZH contributed to the conception and design of the study. Y-FX organized the database and wrote sections of the manuscript. X-DW performed the statistical analysis. W-HZ wrote the first draft of the manuscript. All authors contributed to the article and approved the submitted version.

## Conflict of Interest

The authors declare that the research was conducted in the absence of any commercial or financial relationships that could be construed as a potential conflict of interest.

## Footnotes

1 http://hannonlab.cshl.edu/fastx_toolkit/

2 http://soap.genomics.org.cn/

3 http://www.ccb.jhu.edu/software/hisat

4 http://www.geneontology.org

5 http://www.genome.jp/kegg

6 https://blast.ncbi.nlm.nih.gov/Blast.cgi

7 http://www.bioconductor.org/

8 http://www.bioconductor.org/packages/release/bioc/manuals/edgeR/man/edgeR.pdf

9 http://frodo.wi.mit.edu/

## Supplementary Material

FIGURE S1 A volcano plot of differentially expressed genes. The red and blue points indicate notable up- and down-regulation, respectively. The gray points mean that no genes varied significantly.

TABLE S1 Top 10 most up- and down-regulated differentially expressed genes.

TABLE S2 Primers used in qPCR detection for RNA-seq results confirmation.
