# Supplementary figures and images for: Transcriptome Profile Changes Associated With Heat Shock Reaction in the Entomopathogenic Nematode, Steinernema carpocapsae
Source: Front Physiol. 2020 Jul 10;11:721. doi: 10.3389/fphys.2020.00721 (PMC7365922; doi:10.3389/fphys.2020.00721)

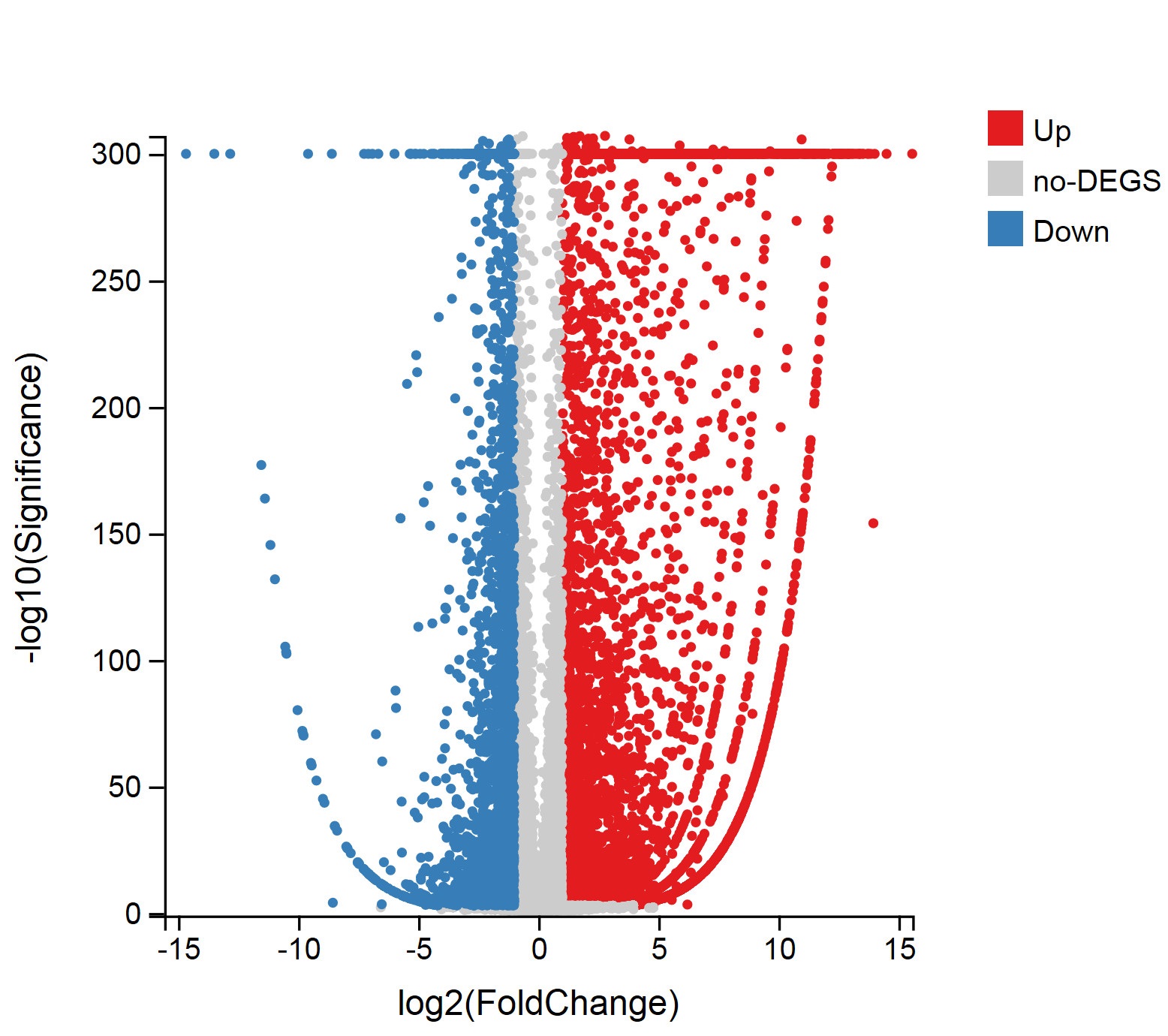

Supplement: FIGURE S1 — A volcano plot of differentially expressed genes. The red and blue points indicate notable up- and down-regulation, respectively. The gray points mean that no genes varied significantly. [file Image_1.JPEG]
