# Supplementary material for: Transcriptome Profile Changes Associated With Heat Shock Reaction in the Entomopathogenic Nematode, Steinernema carpocapsae
Source: Front Physiol. 2020 Jul 10;11:721. doi: 10.3389/fphys.2020.00721 (PMC7365922; doi:10.3389/fphys.2020.00721)
Supplement: TABLE S1 — Top 10 most up- and down-regulated differentially expressed genes. [file Table_1.docx]

**Table S1. Top 10 most up- and down-regulated DEGs.**

| Gene ID | log_2_Ratio  (treatment/control) | P-value | Gene description |
| --- | --- | --- | --- |
| up-regulated genes |  |  |  |
| BGI_novel_G002856 | 15.577 | 0 | -- |
| L596_015286 | 14.509 | 0 | ladderlectin-like |
| L596_020571 | 14.013 | 0 | proline-rich extensin-like protein EPR1 |
| L596_008035 | 13.959 | 9.92E-155 | Synapse-associated protein 1 |
| L596_008536 | 13.848 | 0 | predicted protein |
| L596_025946 | 13.752 | 0 | -- |
| L596_015292 | 13.711 | 0 | low affinity immunoglobulin epsilon Fc receptor |
| L596_026625 | 13.536 | 0 | collagen alpha-1(I) chain-like |
| L596_010364 | 13.524 | 0 | hypothetical protein CRE_18410 |
| L596_008921 | 13.418 | 0 | -- |
| Down-regulated genes |  |  |  |
| L596_007347 | -14.666 | 0 | WD repeat domain 39 |
| L596_009732 | -13.488 | 0 | Decaprenyl-diphosphate synthase subunit 1 |
| L596_006910 | -12.825 | 0 | hypothetical protein Tcan_09608 |
| L596_007836 | -11.530 | 8.96E-178 | Lipase-like protein |
| L596_025081 | -11.379 | 1.71E-164 | hypothetical protein ASU_06948 |
| L596_018750 | -11.150 | 4.07E-146 | insulin-like receptor |
| BGI_novel_G001122 | -10.963 | 1.57E-132 | -- |
| L596_007420 | -10.534 | 7.53E-106 | nematode cuticle collagen domain protein |
| L596_028217 | -10.495 | 1.00E-103 | histone deacetylase 11 |
| BGI_novel_G001197 | -10.483 | 4.76E-103 | -- |
|  |  |  |  |

**Table S2. Primers used in qPCR detection for RNA-seq results confirmation**

| **Gene** | **Primer** |
| --- | --- |
| LIPS17 | F: TGGCTACGAAAGCGAAAACT  R: CAAGTGTCTCCCCAAGTGGT |
| DAF-16 | F: ATTTGGGTGCGAACTTCAAC  R: TCTGGAATCTCCCATTCTGG |
| SOD | F: CAGCTTTTGCTGTCGCTATG  R: ATATCCCCCTTCTGGTGGAC |
| HSP12.2 | F: TGCAGCACAACGATGGTATT  R: AGTTGGGTCGACATCTGAGG |
| HSP12.6 | F: CGCAGTTCTTCACCCCTAAA  R: GGCAGTGATGGAGAGGATTC |
| HSP70 | F: GCTTTCCATGGGAATTGAGA  R: GTTTCCAAGGCGATTGTTGT |
| HSP20 | F: TGAGGCTCGAAATGACAGTG  R: TCCATCTCCTTGGCTTGTTC |
| DAF-2 | F: GGAAGCGTGGCTGAATACAT  R: TCTTCGAAGTTGTCGTCGTG |
| CYP2C | F: TTTGAGAGATTTCGGGATGG  R: AAAAGGCGTTTCCATTTCCT |
| GST | F: GCGAGAGGACAGAGTGGAAC  R: GCTTCTCCTGGTCGTCGTAG |
